# A Comprehensive Study of Chicken Eggs Quality in Short- and Long-Term Storage Under Different Temperatures

**DOI:** 10.3390/foods15162850

**Published:** 2026-08-15

**Authors:** Daniela Bermudez-Aguirre, Joshua Carter, Vijay K. Juneja, Brendan A. Niemira

**Affiliations:** 1 Characterization and Interventions for Foodborne Pathogens, United States Department of Agriculture, Agricultural Research Service, Eastern Regional Research Center, 600 East Mermaid Lane, Wyndmoor, PA 19038, USA; 2 Family and Consumer Sciences, North Carolina Agricultural and Technical State University, 1601 East Market St, Greensboro, NC 27411, USA; 3 Microbial and Chemical Food Safety Research Unit, United States Department of Agriculture, Agricultural Research Service, Eastern Regional Research Center, 600 East Mermaid Lane, Wyndmoor, PA 19038, USA; 4 Institute of Food Technologists, 433 West Van Buren St, Chicago, IL 60607, USA

**Keywords:** poultry, refrigeration, kinetics, shelf-life

## Abstract

There is a lack of regulations worldwide regarding the storage conditions for commercial chicken table eggs. This study was focused on the short (STS–24 days)- and long (LTS–15 weeks) term storage of eggs (*n* = 1500) at three temperatures (10 °C, 21 °C and 35 °C) conducting a comprehensive physical and quality evaluation based on weight loss, Haugh unit (*HU*), yolk index (*YI*), eggshell strength, albumen and yolk pH, yolk color, and albumen absorbance. A zero-order kinetics equation was fitted to the data presenting quality decay, and the half-life time (*t*_1/2_) and decimal reduction time value (*D*) were calculated. Results showed a strong effect of temperature (35 °C) on quality degradation during STS, mainly in weight loss, *HU*, *YI*, yolk color, and absorbance. Refrigeration was able to maintain the quality through the STS and resulted in minor changes during LTS. The net change of color (Δ*E*) and chroma (*C*) of yolk at 35 °C showed significant changes (*p* ≤ 0.05) at the end of STS. The evaluation of *t*_1/2_ showed that eggs stored at 35 °C required <2 days to change quality parameters. The positive effect of refrigeration has been demonstrated in the overall quality of eggs; these results can be used as a starting point to create new guidelines in countries without any regulations for egg storage.

## 1. Introduction

Eggs are one of the most consumed food products worldwide, representing an excellent source of protein and other nutrients. China leads egg production in the global market, followed by Indonesia, India, the USA, Brazil, Mexico and Russia. In 2025, the annual production of eggs in the USA was about 8980.6 million eggs [1]. Meanwhile, in the European Union (EU), the production is expected to go from 6.4 million metric tons of eggs in 2025 to 6.6 million metric tons of eggs in 2035 [2].

The use of refrigeration to store commercial table chicken eggs has generated controversy over the years. Depending on the country, eggs can or cannot be kept under refrigerated conditions. For example, in the USA, the Food Service and Inspection Service (FSIS) mandates eggs to be stored at or below 4.4 °C (40 °F) during the entire production chain without breaking the chilled conditions at any point, including home storage [3]. Meanwhile, in Canada, the Canadian Food Safety Inspection (CFSI) recommends keeping eggs between 10 °C and 13 °C. However, there are several countries, including Mexico, some members of the EU, the Philippines, Australia, New Zealand, Egypt, and Morocco, to mention a few, in which egg refrigeration is voluntary or not required [4,5]. Regarding the shelf-life of eggs, the regulations vary widely depending on the country. In the USA, refrigerated eggs need to be used within 3–5 weeks after the packaging date; in the EU, the best before date should not go beyond 28 days after laying, and in Korea the regulations recommend keeping the eggs between 0 °C and 15 °C without specific market shelf-life [3,6]. In some countries, like India, the regulatory agency Food Safety and Standards Authority of India (FSSAI) establish the consumption of eggs in 2 weeks if the product is kept at room temperature (30 °C) [4] contrasting strongly with the FSIS [3] recommendations regarding eggs at room temperature; if an egg has been left outside the refrigerator, it needs to be consumed in less than 2 h or discarded. Eggs can be found for sale at room temperature in supermarkets in China, France, Mexico, Spain, or Colombia and in street markets in the Philippines and India [4]. The term room temperature is also ambiguous because some countries experience room temperatures of about 25 °C, while tropical countries can reach temperatures between 35 and 40 °C with very high humidity. There is a lack of comprehensive studies in egg quality and physical attributes at higher temperatures (>30 °C) or for extended time. The presence of *Salmonella* spp. in eggs and egg products during foodborne outbreaks has been widely documented worldwide, as well as the effect of temperature on the growth of this pathogen inside the egg contents and on the eggshell surface [4,7,8,9,10]. However, it is not the purpose of this research to discuss the effect of temperature on the growth of this foodborne pathogen in eggs.

There are several factors that can affect egg quality, such as those related to the hen (genetics, age, laying cycle, housing system of the flocks) and other external factors such as temperature, moisture, time, and management system [11,12]. There are specific attributes that eggs need to maintain during storage to meet certain classifications. For example, egg weight, Haugh unit (*HU*), and yolk index (*YI*) are common standards for eggs in the poultry industry used for grading and quality. Egg weight is used in the USDA program to standardize cartons and ensure price fairness; meanwhile, exterior and interior quality attributes are used for grading, with AA the freshest and highest egg quality [13,13,14]. Similar classifications exist in Canada and Japan, and in the EU eggs are graded as A or B [4]. Egg weight is a physical characteristic that decreases over time because of the exchange of moisture (water evaporation) through the highly porous eggshell surface [15]. *HU* is strongly related to the quality of albumen and the egg weight; the higher the *HU*, the higher the quality [16]. Meanwhile, yolk color and clarity of albumen are characteristics often sought by the consumer. Kinetics equations are used not only to quantify changes in food during storage, but also to predict how these physical, chemical, and biological changes will develop over time. Some of these changes are due to chemical reactions or physical processes because of temperature or time [17]. Some of the parameters that are used to estimate the shelf-life of food include the *D* (decimal reduction time) and *z* (thermal resistance coefficient) values, the half-life time (*t*_1/2_), the temperature quotient, among others [4].

Although there are some egg quality studies already published, there are limitations in the tested temperatures, evaluated parameters, shelf-life periods, and a lack of a deep analysis and mathematical modeling of these attributes. Then, the aim of this research is to study the changes in chicken egg physical and quality parameters during short (STS)-and long-term storage (LTS), evaluating the effect of three temperatures on attributes such as egg weight, *HU*, eggshell strength, *YI*, yolk color, pH, and albumen quality. A mathematical model was used to fit the experimental data and estimate some shelf-life parameters. The results from this study highlight the importance of refrigeration in maintaining quality, emphasizing the need to use low temperatures to keep egg quality intact.

## 2. Materials and Methods

### 2.1. Hen Eggs

Two sets of hen eggs were used for this study; the first batch corresponds to large white eggs (grade AA), purchased in a local supermarket in Philadelphia, PA. These eggs were used for the STS study, and several dozen were purchased in different weeks using the same brand from the same supermarket. For the LTS study and for logistical reasons, four large batches of large white eggs (grade AA, 30 doz. eggs each box) were received from a local farm in PA. Based on several previous studies, eggs from a single farm might contain units from different flocks, from different hen ages, and different breeds and are commonly supplied to local supermarkets based on weight per dozen or 30-dozen egg boxes. In both cases, eggs were kept refrigerated (4 °C) and used within the next day. The eggs from the supermarket and from the farm were <7 days old. Each egg was visually checked for cracks on the eggshell, and those with physical damage were discarded. Eggs were individually weighed, and the weight was recorded on the eggshell of each one for further analysis. It is estimated that the total number of eggs analyzed (*n*) for this study was about 1500 units.

### 2.2. Egg Storage

Three temperatures were chosen to conduct the egg storage studies for STS: 10 °C, 21 °C, and 35 °C; for LTS eggs were kept only at 10 °C based on previous results of STS. These temperatures were chosen based on the different scenarios worldwide for storing eggs in commercial facilities such as supermarkets, convenience stores, farmers’ markets, local markets, and street vendors [4]. The refrigeration temperature chosen for the present study (10 °C) does not represent the ideal storage temperature for eggs in the USA (4 °C). So, this temperature can be considered a “refrigeration abuse temperature” in the USA but certainly represents a regular refrigeration temperature in other countries or small markets [4]. Three incubator chambers (MIR-154-PA, Panasonic Healthcare Co., Ltd., Tokyo, Japan) were set at the three temperatures the day before the experiment. For the STS eggs were placed in open egg cartons for up to 24 days. For the LTS eggs were placed in open egg cartons for up to 15 weeks. Each testing day, at least three eggs were taken from each batch and analyzed for quality attributes as described below. Before the analysis, eggs were checked again for any cracks or defects.

### 2.3. Egg Physical and Quality Assessment

Several physical and quality attributes were assessed for each egg during STS and LTS. These characteristics included egg weight, eggshell strength, *HU*, *YI*, eggshell thickness, yolk color, albumen and yolk pH, and albumen absorbance.

#### 2.3.1. Weight

Eggs were retrieved from the incubators and allowed to reach room temperature (21 °C) for a few minutes before being weighed. Each egg was weighed in an analytical balance Ohaus, Pioneer PA 512 (Ohaus, Corp., Parsippany, NJ, USA), and the weight was recorded (±0.01 g).

#### 2.3.2. Eggshell Strength and Thickness, Haugh Unit (HU), and Yolk Index (YI)

For these characteristics, a digital egg tester DET 6500 (Nabel Co., Ltd., Kyoto, Japan) was used. For eggshell strength, eggs were laid horizontally on the equipment compartment, and using low-speed pressing, the strength to crack the eggshell was recorded (±0.2 kg_f_). Next, the egg was cracked in the testing plate of the egg tester, and the *HU* and *YI* were obtained after the testing scan. *HU* is based on the egg weight (±0.1 g) and the height of thick albumen (±0.2 mm). *YI* is the ratio between the yolk height (±0.2 mm) and the yolk diameter (±0.2 mm). Finally, a small piece of the eggshell from the equator of the egg was used to measure the eggshell thickness. The internal membrane was removed, and a gauge meter attached to the egg tester was used to record this measurement (±0.02 mm).

#### 2.3.3. Yolk Color

The yolk color was evaluated at least in three different spots using a colorimeter JZ-300 (Shenzhen Kingwell Instrument, Co., Ltd., Shenzhen, China). The Hunter color parameters, L*, a*, and b*, were measured for each yolk. L* represents the lightness (100) or darkness (0) of the sample; a* represents the redness (+) or greenness (−); and b* represents the yellowness (+) or blueness (−) of the product. The colorimeter has an accuracy of ΔE* < 0.1. Additional color functions were calculated based on L*, a*, and b* as follows. The net color difference (Δ*E*) was evaluated for the yolk samples using the following equation:

(1) $$∆E=\sqrt{{\left(∆L\right)}^{2}+{\left(∆a\right)}^{2}+{\left(∆b\right)}^{2}}$$

where Δ*L* is the difference between the initial and the final L* value, Δ*a* is the difference between the initial and the final a* value, and Δ*b* is the difference between the initial and final b* value inside the same temperature group. Initial values correspond to control samples on day 0.

The chroma saturation index (*C*) was evaluated with Equation (2):

(2) $$C={\left(a^{*2}+b^{*2}\right)}^{1/2}$$

#### 2.3.4. Albumen and Yolk pH

The egg components (i.e., yolk and albumen) were separated manually and placed in individual beakers. The samples were homogenized by hand using a spatula, and then the pH was measured by direct immersion of the electrode (21 °C) using a Mettler Toledo pH meter Seven Compact S220 (Columbus, OH, USA). The pH meter was previously calibrated with three standard buffers (10.01, 7.00, 4.01). The pH of each sample was measured in triplicate (±0.002).

#### 2.3.5. Albumen Absorbance

Albumen absorbance was assessed in the eggs during STS and LTS as an indication of protein degradation in the product because of the effect of temperature and time. The egg albumen was manually separated from the yolk, which was discarded. The egg albumen was homogenized with a magnetic stirrer for 5 min using a hot stirrer plate (500 rpm, no heat). Then, albumen was transferred into test tubes and sonicated in a bath (model 15337402, Fisher Scientific, Pittsburgh, PA, USA) for 5 min to remove bubbles. Samples were transferred to disposable polystyrene UV cuvettes, and the absorbance was read at 600 nm in a spectrophotometer (Model 83057-06, Cole Parmer, East Hills, IL, USA) calibrated with deionized water (±0.004). Three samples were taken from each egg.

### 2.4. Mathematical Modeling

The quality degradation was assessed using a zero-order kinetics equation for those physical and quality attributes that showed changes over the storage period. The equation is expressed as follows:

(3) $$C=C_{0}-kt$$

where *C*_0_ is the initial value of the quality characteristic (*C*), *k* is the rate constant, and *t* is the storage time. The data fitting with the equation was conducted with Microsoft Excel (Version 2501, Seattle, WA, USA).

The half-life time (*t*_1/2_) was also calculated for those parameters showing changes over time using the rate constant (*k*) as follows:

(4) $$t_{1/2}=\frac{ln2}{k}$$

The decimal reduction time (*D*) for quality degradation during the storage was calculated as follows:

(5) $$D=\frac{ln10}{k}$$

where *k* is the rate constant.

### 2.5. Statistical Analysis

All the experiments were conducted at least in triplicate on different weeks with fresh eggs. At least three eggs were used for each measurement every time an experiment was conducted (at least 9 eggs per point); the total number of eggs was 1500 units (*n*). Results are expressed as average values ± standard deviation (Microsoft Excel, Version 2501, Seattle, WA, USA). Furthermore, an analysis of variance (ANOVA-one-way) was calculated using SAS (Version 9.4, Cary, NC, USA) with a confidence level of *α* = 0.05 to determine any significant difference between temperatures. Also, a pairwise Tukey’s test was used to find significant differences between the tested conditions using *α* = 0.05.

## 3. Results and Discussion

This research studied the STS of eggs at three different temperatures, 10 °C, 21 °C, and 35 °C, and the LTS only at refrigerated conditions (10 °C). To facilitate the reading and comparison between STS and LTS, the results will be discussed based on specific quality attributes, highlighting the effect of temperature and time during storage.

### 3.1. Egg Weight

**Figure 1:**
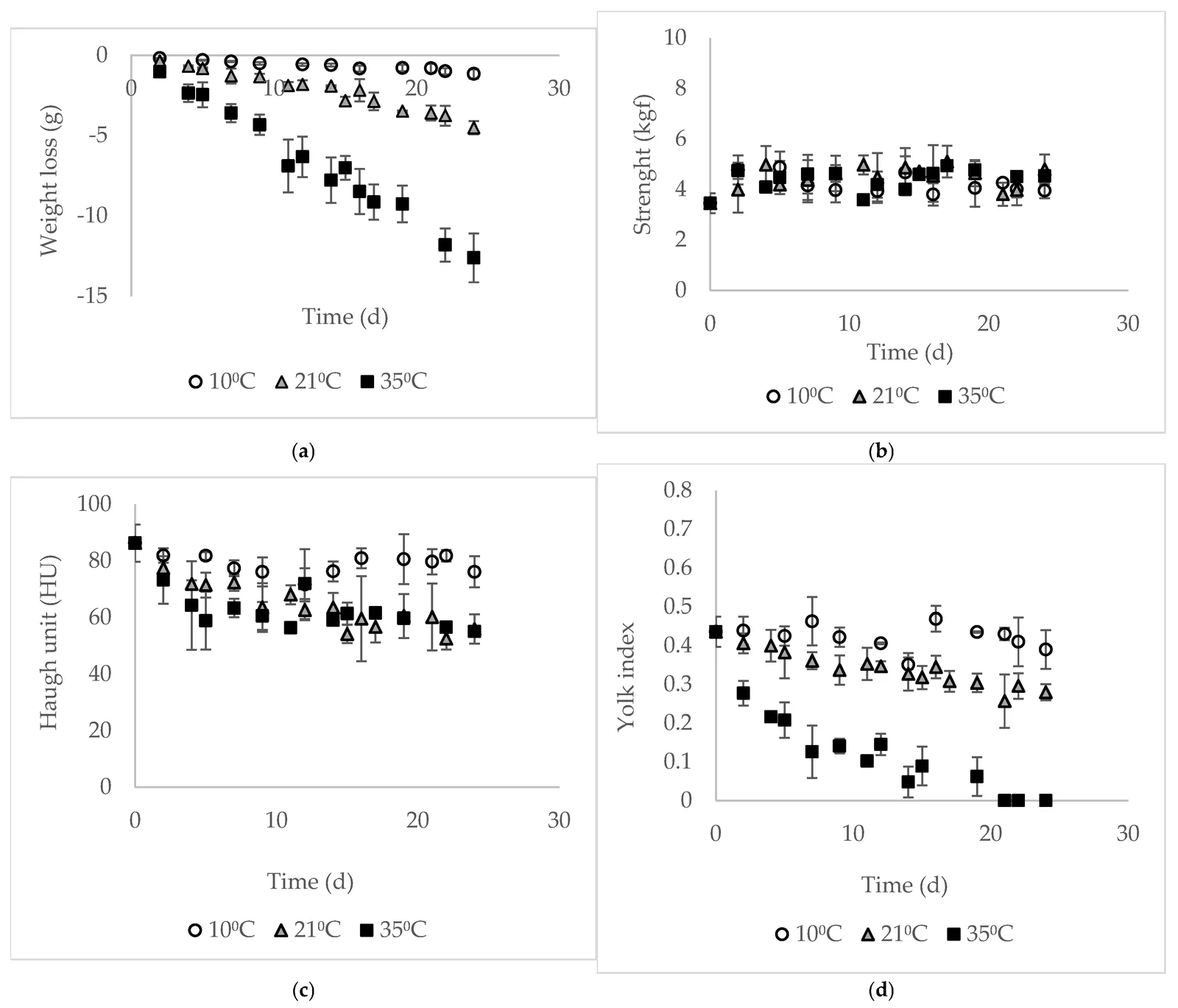
Physical properties of eggs: (**a**) weight loss, (**b**) eggshell strength, (**c**) Haugh unit, (**d**) yolk index, during short-term storage (STS, 24 days) at different temperatures (○ 10 °C, ▲ 21 °C, ■ 35 °C).

**Figure 2:**
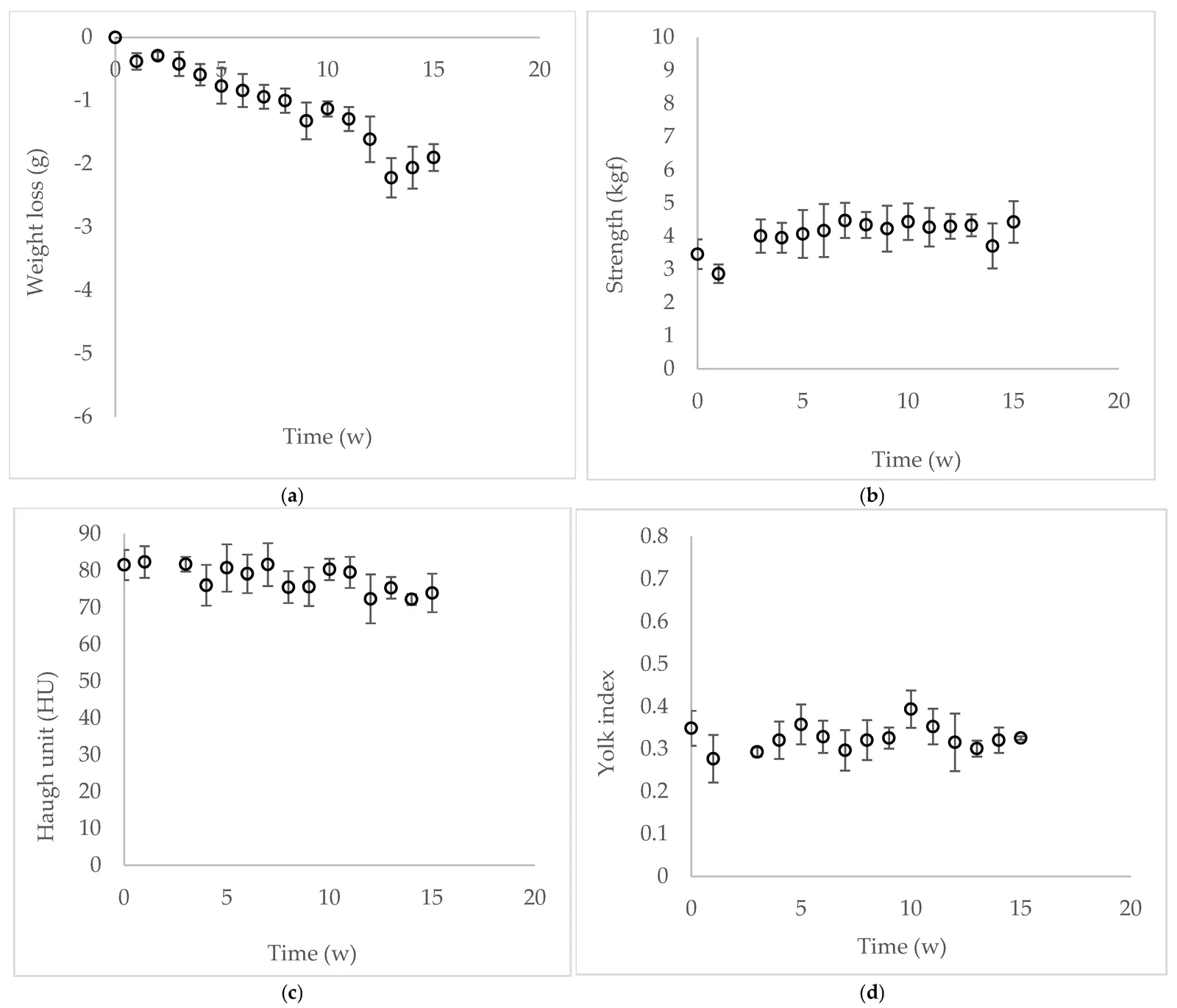
Physical properties of eggs: (**a**) weight loss, (**b**) eggshell strength, (**c**) Haugh unit, (**d**) yolk index, during long-term storage (LTS, 15 weeks) at refrigerated conditions (○ 10 °C).

Egg weight is a critical factor in the egg industry not only for the six standard USDA weight classes, but also for other attributes like *HU*. The results of egg weight loss for this study are presented in Figure 1a for STS and Figure 2a for LTS. The effect of temperature on weight loss was evident for STS; the eggs kept under refrigerated conditions for 24 days showed a minor variation in weight (~1 g, 1.7%). The eggs at 21 °C presented some weight variation through the storage, with a maximum loss of 4.5 g (~7.9%) by the end of the shelf-life. When the temperature was increased to 35 °C, the weight loss reported by day 24 went beyond 12 g (>21%) because of fast water evaporation accelerated by the temperature. During the LTS under refrigerated conditions, the weight remained constant for about 8 weeks, with a further decline, with a maximum weight loss of about 2 g (3.5%) by week 15, as observed in Figure 2a. Egg weight is influenced by the weight, breed, genetics, feed and water, diseases, and age of the hens [12,18]. The standard egg weight is considered between 58 and 62 g, and the laying intensity is another factor influencing this parameter. It is believed that egg laying reaches its peak between weeks 34 and 35 of the hen’s age, producing heavy eggs [12]. As birds age, weight continues to slowly increase until the end of the production cycle (72–74 weeks) [19]. In a reported study about extended storage of eggs at 4 °C and 80% RH, eggs lost about 4 g after 10 weeks of storage for two of the replicates; meanwhile, the third batch of eggs showed a decrease in weight from 65 to 58 g, showing the high variability in egg units [20]. In one of the very few studies conducted at higher temperatures, authors reported egg weight loss of about 4.2% when eggs were stored at 28–31 °C for 28 days, because of water evaporation through the porous structure [21].

### 3.2. Eggshell Strength and Thickness, Haugh Unit (HU), and Yolk Index (YI)

Eggshell strength is also considered an important quality factor because the shell has a vital function in protecting the egg contents from microbial contamination, but also it carries the albumen and yolk to the final use. The eggshell strength results for STS and LTS are presented in Figure 1b and Figure 2b, respectively. There were no significant variations in this parameter regardless of the temperature or storage time, with average values of 4.4 kg_f_. The shell is composed of a crystalline and calcified matrix composed of about 98% CaCO_3_, highly porous (>8000 pores), allowing the exchange of gases and water; high values of shell strength are usually associated with low fragility [15,22]. Shell strength is also correlated to the eggshell thickness, and both parameters are reduced when eggs come from aged flocks because of changes related to calcium metabolism [15]. The standard eggshell strength reported for hen eggs is 3.73 ± 0.74 kg_f_ [11], which is similar to the value found in the present research. In an extended storage study of eggs at 4 °C and 80% RH, eggshell strength did not show differences either after 10 weeks of storage, showing an average value of 3.7 kg_f_ [20].

The eggshell thickness was assessed in all the eggs for STS and LTS, and the values were kept constant over time without important changes. The average eggshell thickness measured for eggs was 0.39 ± 0.01 mm. According to Şekeroğlu and Altuntaş [18], the eggshell thickness is influenced by the size of the egg, showing values of 0.400 mm for medium eggs (~52 g) and going up to 0.387 mm for jumbo eggs (~71 g). The average weight for the eggs used in the present research was about 57 ± 0.5 g, which falls in the range of large eggs (~57 g) reported by the same authors with a shell thickness of 0.386 mm. Monira et al. [23] also noted that breeding is a determining factor influencing eggshell thickness, weight, egg dimensions, albumen height, and *HU*.

The Haugh unit (*HU*) is a standard parameter for evaluating egg quality [24]. In the present research, the *HU* was impacted by the temperature during STS, as shown in Figure 1c, showing a similar decrease for the 25 °C and 35 °C treatments, ending with *HU* close to 55 but keeping high *HU* values for the eggs at 10 °C for STS (80) and LTS (74), as shown in Figure 2c at the end of the shelf-life study. *HU* is directly related to the height of the thick albumen and the egg weight, and it was observed in this research that refrigeration kept these two characteristics very well preserved through time. However, by day 4 at 35 °C, the height of the thick albumen was difficult to measure, and a gradual degradation and thinning process was affected mainly by the temperature and the water transfer from albumen to yolk. The egg weight was also affected when stored at high temperature because of water evaporation. Both changes, thinning of the thick albumen and egg weight decrease, led to a decrease in HU at elevated temperatures. In a different study, eggs were stored at three different temperatures (5, 13 and 23 °C), showing similar *HU* for the eggs at 5 °C after 7 weeks of storage but decreasing *HU* from 72.44 to 59.99 for the eggs at the highest temperature at the end of the shelf-life [24]. In an extended study for egg storage, *HU* went from 82.59 to 67.43 after 10 weeks at 4 °C and 80% RH [20]. However, there is also a low temperature limit at which egg quality is negatively affected; Shin et al. [25] evaluated egg quality at −1.1, 0.6, 2.2, 3.9, 5.6 and 7.2 °C, over 4 weeks. The results showed the highest *HU* at 0.6 and 2.2 °C, but the quality was damaged using lower temperatures. In an early study on the effect of age of flock on *HU*, the results showed that *HU* was higher (94.1) for early lay (<25–40 weeks) and decreased to 81.8 as the hen age increased (>65 weeks) [26]. Meanwhile, in the study previously mentioned using higher temperature (28–31 °C), the *HU* went from 80 to 42 after 28 days [21].

Yolk index (*YI*) in fresh eggs is expected to have a value of about 0.45 and decrease over time [27]. In this study, the *YI* was also affected by the temperature during STS, as shown in Figure 1d, with a fast decline in this characteristic during the first 10 days at 35 °C, ending with values close to zero. During the last days at 35 °C, egg yolks were difficult to handle because of the rupture of internal membranes; by day 20, an unpleasant odor developed inside these eggs. For the 21 °C egg batch, the *YI* showed a slight decrease over storage, ending with values close to 0.260. The effect of refrigeration once again showed a positive effect on preserving and maintaining an almost constant *YI* during STS (~0.400, Figure 1d) and LTS (~0.326, Figure 2d). The egg yolk is surrounded by a thin and transparent membrane known as the vitelline membrane that limits the exchange of materials between yolk and albumen [15]. However, as time increases, the strength of this membrane decreases, allowing the yolk to expand (flatten out) and reducing the *YI.* Heat also has a negative effect on the vitelline membrane, weakening its structure and reducing yolk firmness. High temperature also increases the water transfer from the albumen to the yolk [20,28]. In the work published by Keener et al. [24], *YI* showed minimal changes according to the temperature (5, 13 and 23 °C) after 7 weeks of storage. In a very early study conducted in Kuwait at extreme temperatures such as 37 and 42 °C, simulating local temperatures (45 ± 3 °C), the *YI* was quantified only after 12 days of storage with very low values (0.10), like the ones observed at 35 °C in the present work. No further evaluations were conducted in that early study due to egg quality deterioration caused by exposure to extreme temperatures [28].

### 3.3. Albumen and Yolk pH

**Figure 3:**
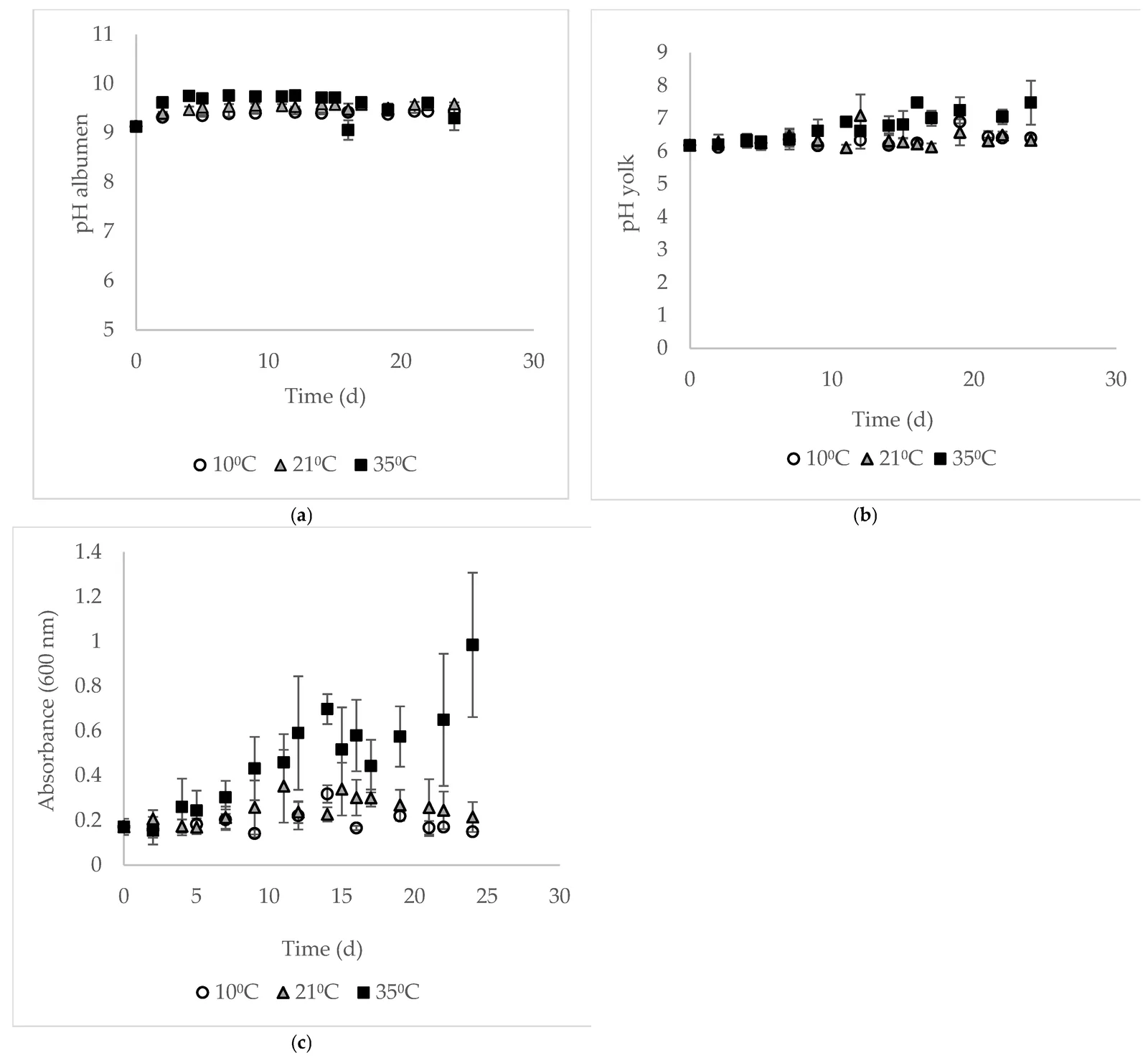
Physical-chemical properties of eggs: (**a**) pH albumen, (**b**) pH yolk, (**c**) absorbance during short-term storage (STS, 24 days) at different temperatures (○ 10 °C, ▲ 21 °C, ■ 35 °C).

**Figure 4:**
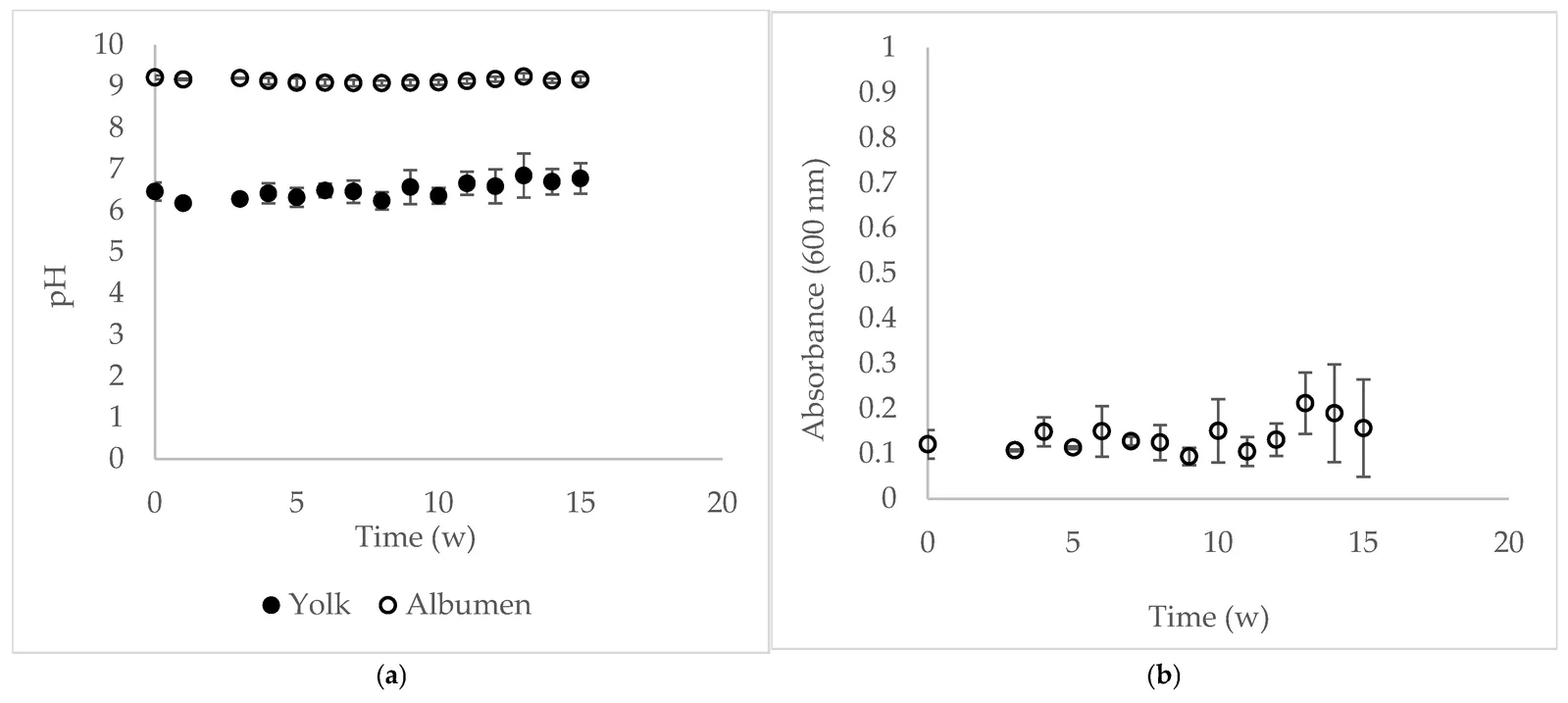
Physical-chemical properties of eggs: (**a**) pH yolk and pH albumen, (**b**) absorbance during long-term storage (LTS, 15 weeks) at refrigerated temperature (○ 10 °C).

pH is a physicochemical characteristic that can provide information regarding chemical changes happening in the product during storage. The changes in pH during STS are presented in Figure 3a,b for albumen and yolk, respectively. While the pH of the albumen remained constant (~9.5) during storage without effect of the temperature, the changes in the pH of the yolk were more evident at the end of STS (day 24) at 35 °C with an increase in value from 6.18 to 7.48. Again, these changes at high temperatures can be because of degradation of internal egg structures like membranes and components such as proteins. Meanwhile, during LTS (Figure 4a), the pH of the albumen remained constant, and again the pH of the yolk showed a slight increase of only 0.3 units. Duman et al. [29] reported pH values for fresh egg albumen from 8.67 to 8.70 depending on the shape index (ratio between width and length of an egg). Meanwhile, Scott and Silversides [30] reported an increase in the albumen pH of eggs stored at 20.2 °C for 10 days, increasing from 7.34 to 9.37. Lee et al. [6] mentioned a significant increase in albumen and yolk pH as the storage time and temperature increased. Albumen pH went from 7.6 to 9 at 25 °C, and the yolk pH ranged from 5.6 to 6.3 (25 °C) after 30 days. In a different study using only refrigerated temperatures (<7.2 °C), the albumen pH showed only minor changes during storage (28 days), increasing from 8.65 to 9.19 due to the diffusion of gas inside the eggs [25]. In general terms, the albumen alkalinity is related to low egg quality; using refrigerated temperatures reduces the CO_2_ loss from the inside of the eggs and preserves the overall quality [27].

### 3.4. Albumen Absorbance

Egg albumen contains water (88%), proteins (11%), minerals and carbohydrates (1%), and it has been demonstrated that eight essential amino acids (Lys, Try, Phe, Met, Thr, Ile, Leu, Val) are present in this egg component. Albumen characteristics can be influenced by genetics, pH, storage time, and dry matter [11]. The albumen of a fresh egg typically has an absorbance value close to 0.100. As this value increases, the albumen presents some turbidity, indicating the transfer of water and other compounds among egg components as well as some degree of protein denaturation. During STS, albumen absorbance showed a significant increase by the end of the shelf-life when eggs were stored at 35 °C, with values higher than 1.0 as shown in Figure 3c. Meanwhile, for 10 °C and 21 °C, the absorbance remained almost constant. During the LTS, albumen absorbance showed a slight increase by week 13, moving from the initial value of 0.121 (±0.032) to a final value close to 0.200, as observed in Figure 4b. During storage, albumen denaturation occurs, turning this gel-like compound into a watery substance [22]. As the albumen moves to higher values of absorbance, the functionality of some of the proteins present in this egg component can be negatively affected (i.e., gelation, foaming, aeration). These high values were observed for eggs at 35 °C with a fast development after the first 10 days of storage.

### 3.5. Egg Yolk Color

**Figure 5:**
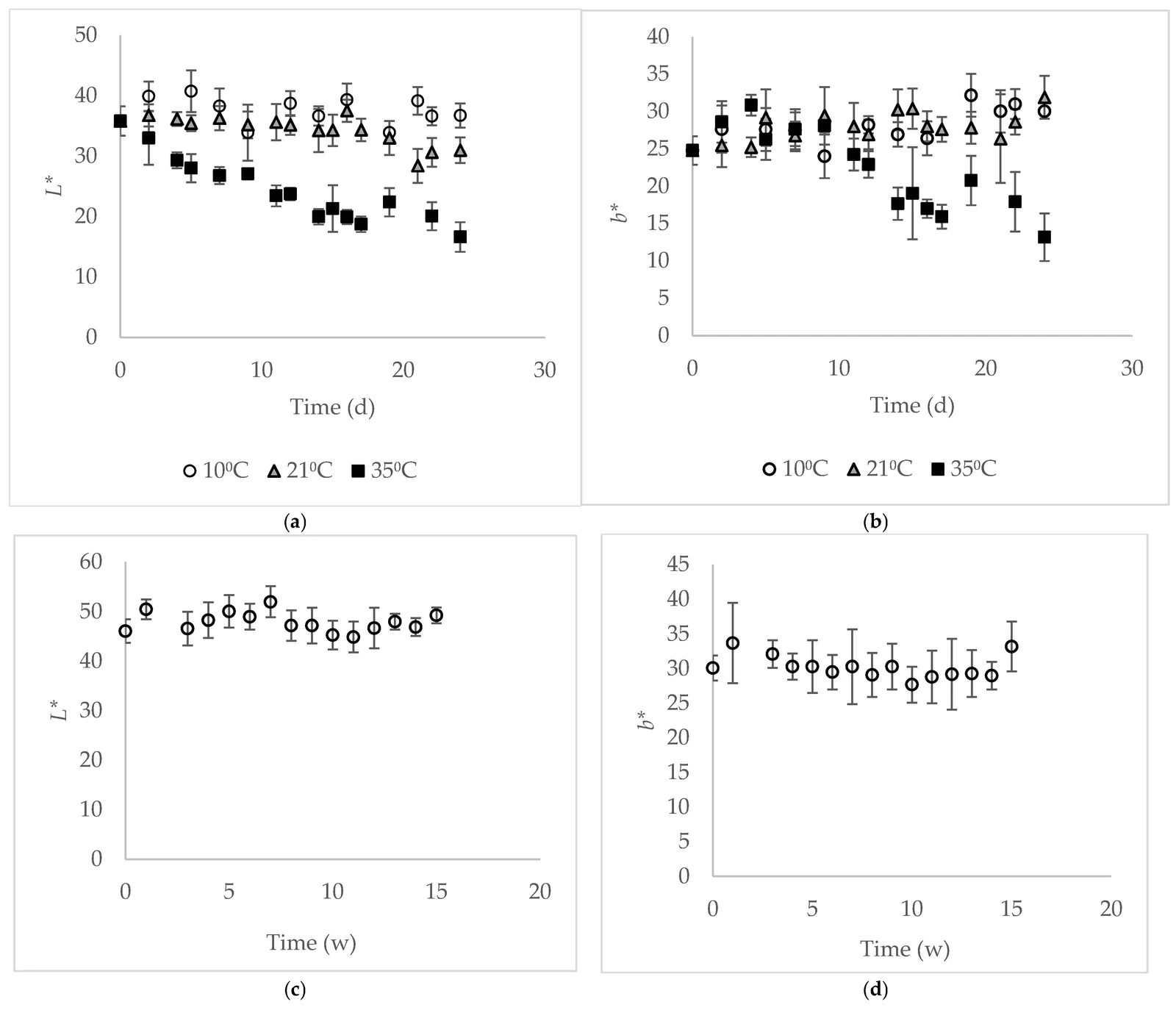
Hunter color parameters, L* and b*, in egg yolk during short-term storage (**a**,**b**) (STS, 24 days) at three different temperatures (○ 10 °C, ▲ 21 °C, ■ 35 °C) and during long-term storage (**c**,**d**) (LTS, 15 weeks) at refrigerated conditions (○ 10 °C).

The yolk color is often a characteristic that consumers relate to high or low quality of eggs. Depending on the country, consumers are looking for a specific yolk color, and it can be modified with the hen’s diet. The ingestion of yellow-orange pigments such as xanthophylls from diverse plants can be deposited in the yolk [31]. There are several scales to measure yolk color, such as the Roche color fan, the CIE scale, and the Hunter (L* a* b*) color scale. In this work, the Hunter color scale was used to evaluate the possible changes during storage. Although the three Hunter parameters, L*, a*, and b*, were measured for each yolk, only L* and b* are presented in Figure 5. However, the three parameters were used to calculate the color functions in Table 1. L* is related to the luminosity of the sample, while b* is related to the yellowness of the sample. In Figure 5a, L* is presented for STS, and there is an important decrease in the luminosity of the yolk as time progresses when the temperature is 35 °C. Similar behavior was observed for the b* parameter during STS (Figure 5b). The shift of the b* value to lower values indicates a loss of yellowness in the sample. Meanwhile, the L* and b* values for LTS remained constant during the 15 weeks with minor variations through the storage. The net change of color, Δ*E*, was calculated for each set of eggs during STS, and the results showed significant differences (*p* ≤ 0.05) for the eggs at the highest temperature (35 °C) when compared to 10 °C and 21 °C. The highest Δ*E* was 22.73 (35 °C), whereas Δ*E* values for 10 °C and 21 °C were equal (Tukey’s test α = 0.05). The color function chroma (*C*) also showed significant differences for 35 °C at the end of the storage (24 days). However, the *C* was equal for the eggs stored at 10 °C for 15 weeks. A low value of *C* represents a weak or muted color, and a high value is more related to vivid and intense color. During the storage of eggs, the water from albumen crosses the vitelline membrane and enters the yolk, dissolving some of the pigments and decreasing the yolk color [22]. These changes in the vitelline membrane are related to a lack of strength because of aging and chemical changes that also allow the passing of microorganisms from albumen to the rich environment in the yolk [20,24]. Eggs at 35 °C were the most affected in color parameters because of the fast degradation of the pigments present in the yolk.

**Figure 6:**
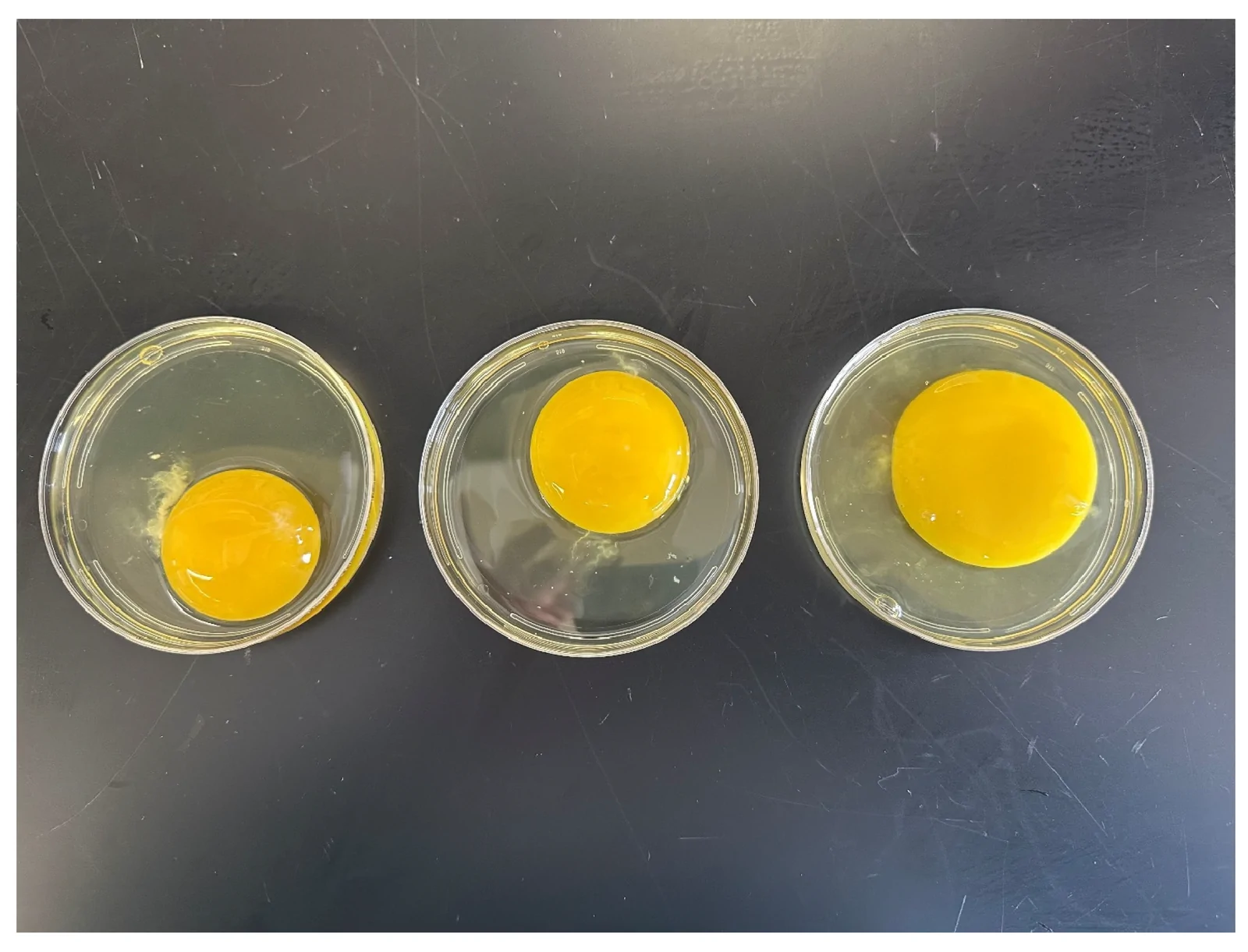
Egg quality appearance at three different temperatures: 10 °C (**left**), 21 °C (**middle**) and 35 °C (**right**) on day 15 of storage.

The appearance of the eggs at three different storage temperatures on day 15 is shown in Figure 6. There is a clear difference between the appearance of the egg at 35 °C and the eggs at lower temperatures. The egg yolk looks bigger and flatter; the color has lost its intensity, there is some turbidity in the albumen, and in general, the egg does not look fresh anymore. The albumen has an inner liquid layer that is in contact with the egg yolk and the thick layer that connects to the eggshell. These layers experience changes over time [15]. After day 15, major changes occurred for the eggs at 35 °C; some experienced liquefaction of internal contents, an unpleasant internal odor, and a few more presented breakages of internal membranes when cracked for evaluation. Because of these reasons, eggs at 35 °C were not candidates for long-term storage studies. But the use of refrigeration can delay the negative changes in egg quality over time. Although eggs are considered a fresh product that will be consumed in a few days after purchase, there are still remote locations in which eggs need to be transported from major cities to small towns. The use of refrigeration during egg distribution can also maintain egg quality acceptable for the consumer.

### 3.6. Mathematical Modeling

The quality attributes that showed the greatest variation during storage were the eggs stored at 21 °C and 35 °C, mainly weight loss, *HU*, *YI*, absorbance, and the color parameters L* and b*. Therefore, mathematical fitting was conducted on the shelf-life values using a zero-order kinetic equation. The rate constant (*k*) and the correlation coefficients (*R*^2^) are presented in Table 2 together with half-life time (*t*_1/2_) and the decimal reduction time (*D*). In general, the mathematical fitting was better for the data at 35 °C, showing higher correlation coefficients (*R*^2^). The rate constant (*k*) showed a faster degradation process when the eggs were stored at 35 °C compared to 21 °C. However, 21 °C also showed some changes in egg quality. In a previous study modeling the quality of control eggs and radio frequency processed eggs stored at 7.2 °C, zero-order and first-order kinetics showed good fitting when the models were adjusted to the data from weight loss and *HU* [32]. Regarding the half-life time (*t*_1/2_), which represents the required time to degrade 50% of the quality attribute, results for 35 °C show clearly the effect on weight loss, *HU*, and color. All of them require less than 1.5 days to degrade the quality by half. For the eggs stored at 21 °C, the most affected quality parameters were again weight loss, *HU*, and color, but the required time is about 4 days. Finally, the *D* value or decimal reduction time for quality degradation is an average of 3.6 days at 35 °C and 9.7 days for the eggs stored at 21 °C. In the previously mentioned study, *t*_1/2_ for control eggs in refrigeration was reported as 25.7 weeks for weight loss and 24.15 weeks for *HU*, while the corresponding *D* values were 85.3 weeks and 80.2 weeks for the same quality attributes, respectively [32], showing the positive effect of refrigeration on egg quality.

## 4. Conclusions

The positive effect of temperature has been demonstrated in several egg physical and quality attributes, showing that refrigeration can delay the quality decay for up to 15 weeks. The meaning of “room temperature” is relative to the local area in which eggs are stored, but mild temperatures such as 25 °C still have a negative effect on egg quality. The highest temperature evaluated in this research (35 °C) showed negative impacts on the overall physical and quality attributes of eggs after the first days of storage. Some of the chemical changes happening in the egg components, such as whites and yolk, are not only changing physicochemical values such as pH or absorbance but might affect the nutritional content of the eggs and their functionality. More comprehensive, clearer, and accurate regulations are required worldwide for those countries in which refrigeration of eggs is not mandatory, based on scientific evidence supporting the positive effect of refrigeration on egg quality, together with the food safety aspect.

## Figures and Tables

**Table 1:** Color functions, Δ*E* and *Chroma* (*C*), for the egg yolk stored during short-term at three different temperatures and during long-term at refrigerated conditions.

Short-Term Storage (STS)	10 °C	21 °C	35 °C
Δ*E* (end of storage)	6.49 ^a^ (±1.59)	9.40 ^a^ (±1.12)	22.73 (±0.52)
*C* (initial)	24.97 ^a^ (±1.90)	24.97 ^a^ (±1.90)	24.97 ^a^ (±1.90)
*C* (final)	30.73 ^a^ (±0.01)	32.60 ^a^ (±2.93)	14.66 (±3.32)
**Long** **-** **term storage (LTS)**			
Δ*E* (end of storage)	5.14 (±0.94)		
*C* (initial)	30.21 ^A^ (±1.77)		
*C* (final)	33.40 ^A^ (±3.79)		

Δ*E*: Net change of color. STS: Short-term storage (24 days). LTS: Long-term storage (15 weeks). Average values ± standard deviation. ^a^ Equal lowercase letters indicate statistically equal means (Tukey’s test α = 0.05) when compared between different temperatures. ^A^ Equal uppercase letters indicate statistically equal means (Tukey’s test α = 0.05) when compared at the same temperature between initial and final time.

**Table 2:** Zero-order kinetics constant and quality degradation parameters for physical and quality characteristics of eggs during short-term storage (STS) at 21 °C and 35 °C.

Quality Characteristic	21 °C				35 °C			
	***k*** **(day^−1^)**	***R*** **^2^**	***t*** **_1/2_ (day^−1^)**	***D*** **(day^−1^)**	***k*** **(day^−1^)**	***R*** **^2^**	***t*** **_1/2_ (day^−1^)**	***D*** **(day^−1^)**
**Weight loss**	−0.1785	0.9595	3.88	12.90	−0.5199	0.9812	1.33	4.43
**Haugh unit**	−1.1222	0.8184	0.62	2.05	−0.7624	0.4524	0.91	3.02
**Yolk index**	−0.0062	0.9063	111.79	371.38	−0.0143	0.8249	48.47	161.02
**Absorbance**	0.0037	0.2373	187.33	622.32	0.0279	0.8152	24.84	82.53
L*	−0.2514	0.5660	2.75	9.16	−0.7054	0.8769	0.98	3.26
b*	0.1553	0.3313	4.46	14.83	−0.6384	0.7398	1.08	3.61

*k*: rate constant, *R*^2^: correlation coefficient, *t*_1/2_: half-life time, *D*: decimal reduction time. STS: Short-term storage (24 days).

## Data Availability

The original contributions presented in this study are included in the article. Further inquiries can be directed to the corresponding author.

## Abbreviations

The following abbreviations are used in this manuscript:

*C*	Chroma
CIE	Commision Internationale de l’éclairage (International Commission on Illumination)
CFSI	Canadian Food Safety Inspection
D	D value or decimal reduction time
Δ*E*	Net color change
doz.	dozen
EU	European Union
FSIS	Food Safety and Inspection Service
FSSAI	Food Safety and Standards Authority of India
HU	Haugh unit
Ile	Isoleucine
*k*	rate constant
kg_f_	Kilogram-force
Leu	Leucine
Lys	Lysine
LTS	Long-term storage
Met	Methionine
nm	nanometer
Phe	Phenylalanine
R^2^	Correlation coefficient
rpm	revolutions per minute
STS	Short-term storage
t_1/2_	Half-life time
Thr	Threonine
Try	Tryptophan
USA	United States of America
USDA	United States Department of Agriculture
Val	Valine
YI	Yolk index
